# Nilotinib-Induced Unilateral Renal Artery Stenosis: A Complication Prompting Treatment-Free Remission in Chronic Myeloid Leukemia

**DOI:** 10.1155/crom/9887771

**Published:** 2025-09-08

**Authors:** Mitchell C. Boshkos, Humberto R. Nieves-Jiménez, Parin H. Thakkar, Juan J. Cintrón-García, Omar Mamlouk

**Affiliations:** ^1^Department of Internal Medicine, The University of Texas MD Anderson Cancer Center, Houston, Texas, USA; ^2^Department of Internal Medicine, Baylor College of Medicine, Houston, Texas, USA

## Abstract

Renal artery stenosis (RAS) is a rare but significant vascular complication associated with nilotinib therapy for chronic myeloid leukemia (CML). We present the case of a woman in her mid-70s on long-term nilotinib who developed this adverse event. The patient presented with a progressive, insidious decline in renal function over several years. Diagnostic evaluation revealed severe unilateral stenosis of the left renal artery. Under nilotinib, the patient had maintained a sustained deep molecular response (DMR), making her a candidate for treatment-free remission (TFR). The development of RAS prompted the discontinuation of nilotinib, both as a therapeutic intervention for her kidney disease and to initiate a trial of TFR. Following discontinuation, the patient's renal function showed partial but significant improvement, suggesting a causal relationship. This case describes the importance of recognizing subtle presentations of TKI-induced vascular complications, particularly unilateral RAS, and illustrates how managing such adverse events intersects with modern CML therapeutic goals like TFR.

## 1. Introduction

Tyrosine kinase inhibitors (TKIs) targeting the BCR::ABL1 oncoprotein are the standard of care for chronic myeloid leukemia (CML) [1, 2]. Despite their efficacy, TKIs are associated with a range of adverse events [3, 4], including well-documented renal toxicities like acute tubular necrosis [5], interstitial nephritis [6, 7], and proteinuria [4, 8]. Significant vascular complications are less common, with TKI-induced renal artery stenosis (RAS) being infrequently reported [9, 10] and unilateral presentations being particularly rare. We describe a case of progressive chronic kidney disease (CKD) from insidious, unilateral RAS attributed to long-term nilotinib therapy, highlighting the diagnostic challenge and management implications for a patient pursuing treatment-free remission (TFR).

## 2. Case Presentation

A female patient in her mid-70s with a past medical history of well-controlled hypertension and dyslipidemia was diagnosed with BCR::ABL1-positive CML in 2014. Prior to initiating any therapy, her renal function was normal, with a baseline estimated glomerular filtration rate (eGFR) of approximately 75 mL/min/1.73 m^2^ and a serum creatinine (sCr) of 0.8 mg/dL. She was initially started on imatinib but developed significant gastrointestinal intolerance. Consequently, she was switched to the second-generation TKI, nilotinib. Her hypertension and dyslipidemia were managed concurrently with losartan and rosuvastatin, respectively.

Under nilotinib, she achieved a major molecular response (MMR) within the first year and subsequently maintained a stable deep molecular response (DMR; MR with a BCR::ABL1/ABL1 ratio < 0.0032%) for over 5 years.

In 2016, 2 years into therapy, she was noted to have Stage 3A CKD, with an eGFR of 50 mL/min/1.73 m^2^ (sCr 1.0 mg/dL). Her renal function remained relatively stable until 2021, when her sCr increased to 1.51 mg/dL (eGFR 35 mL/min/1.73 m^2^). By 2023, she experienced a further decline, with an sCr of 1.69 mg/dL and an eGFR of 31 mL/min/1.73 m^2^.

This progressive decline prompted a focused workup. A renal Doppler ultrasound in 2023 revealed that the left kidney had decreased in size to 7.7 cm from 9.1 cm (measured in 2021) and demonstrated a tardus parvus waveform, suggestive of significant upstream stenosis. A subsequent magnetic resonance angiography (MRA) confirmed severe stenosis in the proximal left renal artery. The right kidney and renal artery appeared normal.

Given her sustained DMR and the diagnosis of nilotinib-induced unilateral RAS, the decision was made in early 2024 to discontinue nilotinib and attempt TFR. The patient was started on low-dose aspirin (81 mg daily). Over the following 2 months, her renal function showed notable improvement: her sCr decreased to 1.34 mg/dL, and her eGFR recovered to 41.7 mL/min/1.73 m^2^. Her CML has remained in DMR off therapy, and she continues to be monitored with regular BCR::ABL1 quantitative PCR testing.

## 3. Discussion

This case describes an uncommon vascular adverse event of nilotinib and is particularly instructive for several reasons. First, it describes the insidious nature of unilateral RAS, which can pose a significant diagnostic challenge. The gradual decline in renal function observed over years could easily be misattributed to age-related changes or comorbid hypertension. Our case adds a more novel perspective to the existing literature; for instance, the report by Kristensen et al. described a patient with bilateral RAS [10], which presents in the published literature with more pronounced clinical signs such as refractory hypertension. In contrast, our patient's unilateral disease caused a subtler clinical picture, emphasizing the need for a high index of suspicion in any nilotinib-treated patient with unexplained GFR decline.

Second, this case is framed within the modern context of CML management, where TFR is a primary therapeutic goal for eligible patients [2]. The development of a significant long-term toxicity, such as RAS, directly influences the risk-benefit calculation of continuing TKI therapy in a patient who has already achieved a sustained DMR. Here, the toxicity provided a clear impetus to discontinue the drug, aligning the management of the adverse event with the pursuit of TFR. This highlights the importance of proactive surveillance for long-term toxicities, as they may become critical factors in deciding to stop therapy.

The proposed mechanism of nilotinib-induced vasculopathy involves off-target kinase inhibition, which promotes endothelial dysfunction and inflammation [11]. The partial but significant recovery of renal function after nilotinib withdrawal strongly supports a causal link and underscores the potential for reversibility [9]. Our management strategy—drug cessation, antiplatelet therapy, and continued ARB use—proved effective without requiring invasive revascularization. This conservative approach should be considered as a first-line option, especially when clinical improvement is observed after drug withdrawal.

In conclusion, clinicians must be vigilant for nilotinib-associated vascular complications, including unilateral RAS, which can manifest as an insidious decline in renal function. Early imaging-based diagnosis and prompt discontinuation of the drug can prevent irreversible kidney damage. Further, in CML patients with sustained DMR, the presence of such toxicities should prompt consideration of a TFR attempt, aligning safety concerns with long-term therapeutic goals.

## Data Availability

The data that support the findings of this study are available from the corresponding author upon reasonable request. Due to patient confidentiality and privacy regulations, some data may be anonymized or withheld.

## General Statement

*Patient Perspective*. The authors have nothing to report.
